# Laparoscopic and open gastrectomy for locally advanced gastric cancer: a retrospective analysis in Colombia

**DOI:** 10.1186/s12893-023-01901-2

**Published:** 2023-01-26

**Authors:** Maritza Romero-peña, Liliana Suarez, Diego Efraín Valbuena, Carlos Eduardo Rey Chaves, Danny Conde Monroy, Raúl Guevara

**Affiliations:** 1 School of Medicine, Universidad Sanitas, Bogotá D.C, 110111 Colombia; 2 Clínica Universitaria Colombia, Bogotá D.C, 110111 Colombia; 3 School of Medicine, Pontificia Universidad Javeriana, Carrera 6A# 51A - 48, Bogotá D.C, 110111 Colombia; 4 Hospital Universitario Mayor - Méderi, Universidad del Rosario, Bogotá D.C, 110111 Colombia

**Keywords:** Laparoscopy, Gastrectomy, Advanced gastric cancer, Survival

## Abstract

**Introduction:**

Radical gastrectomy has traditionally been the pillar treatment with curative intent for malignant tumors of the stomach. The safety of the laparoscopic approach for advanced gastric cancer (AGC) is still under debate. In our institution, laparoscopic gastrectomy is the most performed approach.

**Objective:**

Our aim is to describe the experience of a high-volume center in the treatment of AGC in Colombia and to analyze the short-term results and the overall survival rate at 1, 3, and 5 years comparing the open and laparoscopic approaches.

**Methods:**

A cross-sectional retrospective study of patients who underwent gastrectomy for advanced gastric cancer by open or laparoscopic approaches were performed. A Will-Coxon Mann Whitney test was performed in terms of lymph node status and surgical approach. Survival analysis was performed using the Kaplan–Meier method for overall survival at 1, 3, and 5 years. An initial log-rank test was performed to test the relationships between the operative variables and overall survival, the statistical value was accepted if p < 0.20. Data with an initial statistical relationship in the log-rank test were included in a secondary analysis using multivariate Cox proportional regression, variables with a value of p < 0.05 were considered statistically significant.

**Results:**

310 patients met the inclusion criteria. 89% underwent laparoscopic gastrectomy and 10.9% open gastrectomy. The resection margins were negative at 93.5% and the In terms of lymph node dissection, the median lymph nodes extracted was 20 (12;37), with statistically significant differences between the approaches in favor of the laparoscopic approach (Median 21 vs 12; z = − 2.19, p = 0.02). The survival rate was at 1, 3, and 5 years of 84.04%, 66.9%, and 65.47% respectively. The presence of complications and the ICU requirement have a negative impact on survival at 1 year (p 0.00).

**Conclusion:**

A laparoscopic approach is safe with acceptable morbidity and mortality rates for treating gastric cancer. D2 Lymphadenectomy could be performed successfully in a laparoscopic approach in a high-volume center and a properly standardized technique. Major postoperative morbidity with intensive care unit requirement seems to influence overall survival rates.

## Background

Gastric cancer (GC) is the sixth most prevalent malignancy and the third cause of mortality related to oncologic conditions [1, 2]. Approximately 990.000 patients are diagnosed with GC each year, and the incidence seems to increase each year [1–3]. Asia and East Europe have the highest incidence rates compared with North America [4]. Gastric cancer can be divided into early and advanced stages. The early stage is limited to mucosa or submucosa, regardless of the size of the lesion or nodal compromise. Advanced Gastric Cancer (AGC) includes intermediate and advanced tumors (tumors that extend beyond the submucosa). Surgery and chemotherapy are the pillars of treatment, the 5-year survival rate for early GC is 90%. However, the detection rate is low and most patients develop advanced-stage disease (70%) [5]. According to Globocan, at least six south-American countries have the highest prevalence of gastric cancer around the world, and in addition, mortality rates are higher in the Latin-American population in comparison to the United States (18% vs 4%) [4, 6]. Nevertheless, the data on the Latin-American population is still poor [4, 6].

Open radical gastrectomy (OG) was considered for several years the gold standard surgical treatment for GC [7]. The appropriate radical resection includes a complete tumor resection (R0) and a D2 lymph node dissection [8].

However, since the first description by Kitano et al. [9] in 1994 of laparoscopic gastrectomy (LG) for a patient with early gastric cancer; the laparoscopic approach has been popularized [10–12]. Some of the obvious advantages of LG are the minimally invasive approach, less intraoperative blood loss, and a fast recovery [10–12]; however, the positive results depend on the surgeon's expertise, adequate selection of each patient, and management by a multidisciplinary group in a specialized center [10–12]. 

Despite the advantages of LG in the surgical treatment of gastric cancer, minimally invasive techniques remain controversial for the treatment of AGC because of concerns about the adequacy of surgical resection and adequate lymph node dissection [8].

Some studies evidence a decreased rate of harvested lymph nodes comparing laparoscopic versus open approach in patients with AGC and thus represent a limitation of the minimally invasive approach [13]. Other concerning variables are the morbidity rate, hospital length of stay, survival outcomes, and mortality [10–13]. The literature is controversial and there is still a debate on the approach preferences to improve short-term and oncologic long-term survival outcomes [7, 10–13].

The aim of this study is to describe the experience of a high-volume center in the treatment of AGC in Colombia and to evaluate the short-term outcomes and 5-year overall survival rate comparing open and laparoscopic approaches.

## Methods

A cross-sectional study was performed in an institution considered a 4th level hospital in Colombia; with a mean of 14.500 procedures per year, and 80 gastrectomies per year. Is a reference oncologic center in our city.

With institutional board and ethical committee approval. A retrospective review of a prospectively collected database was conducted. All patients over 18 years who underwent gastrectomy for resectable advanced gastric cancer between January 2012 and December 2020 were included. Patients with missing data (follow-up, histopathological reports) were excluded. Ethical compliance with the Helsinki Declaration, current legislation on research (Colombia), and the International Committee of Medical Journal Editors (ICMJE).

Variables included demographic’s characteristics such as age and gender; operative variables among preoperative neoadjuvant therapy, surgical approach, type of gastrectomy and conversion rate, postoperative outcomes were included as well such as morbidity rate, type of complication, intensive care unit requirement and in-hospital stay. Pathological reports were analyzed, and total lymph node retrieval, positivity rate, and surgical margins were evaluated. Overall survival was estimated and defined from the day of the surgery to February 2022 according to the national database reports. Disease-free survival wasn’t included in our analysis due to administrative issues, and we can’t achieve institutional follow-up for all patients.

### Statistical analysis

Descriptive statistics were reported in terms of the variable nature. Qualitative analysis was performed in terms of frequencies and percentages while quantitative analysis was done in terms of mean, standard deviations or medians, and interquartile ranges (IQRs) according to the type of data distribution.

A mean comparison was performed between total lymph node retrieval and harvested lymph nodes in mortality groups using a two-way student T-test or Wilcoxon-Mann–Whitney when appropriate, values of p < 0.05 were considered significant.

Survival analysis was performed using the Kaplan–Meier method for overall survival for 12, 24, and 60 months. An initial log-rank test was performed to prove relationships between operative variables and overall survival, statistical value was accepted if p < 0.20. Data with an initial statistical relationship in the log-Rank test was included in a secondary analysis using multivariate cox-proportional regression; variables with a p-value < 0.05 were considered statistically significant. Statistical analysis was performed using the Statistical Package of STATA Version 17.0 BE-Basic Edition (StataCorp LLC StataCorp 4905 Lakeway Drive College Station, Texas 77845 USA).

### Surgical approach and management

**Fig. 1:**
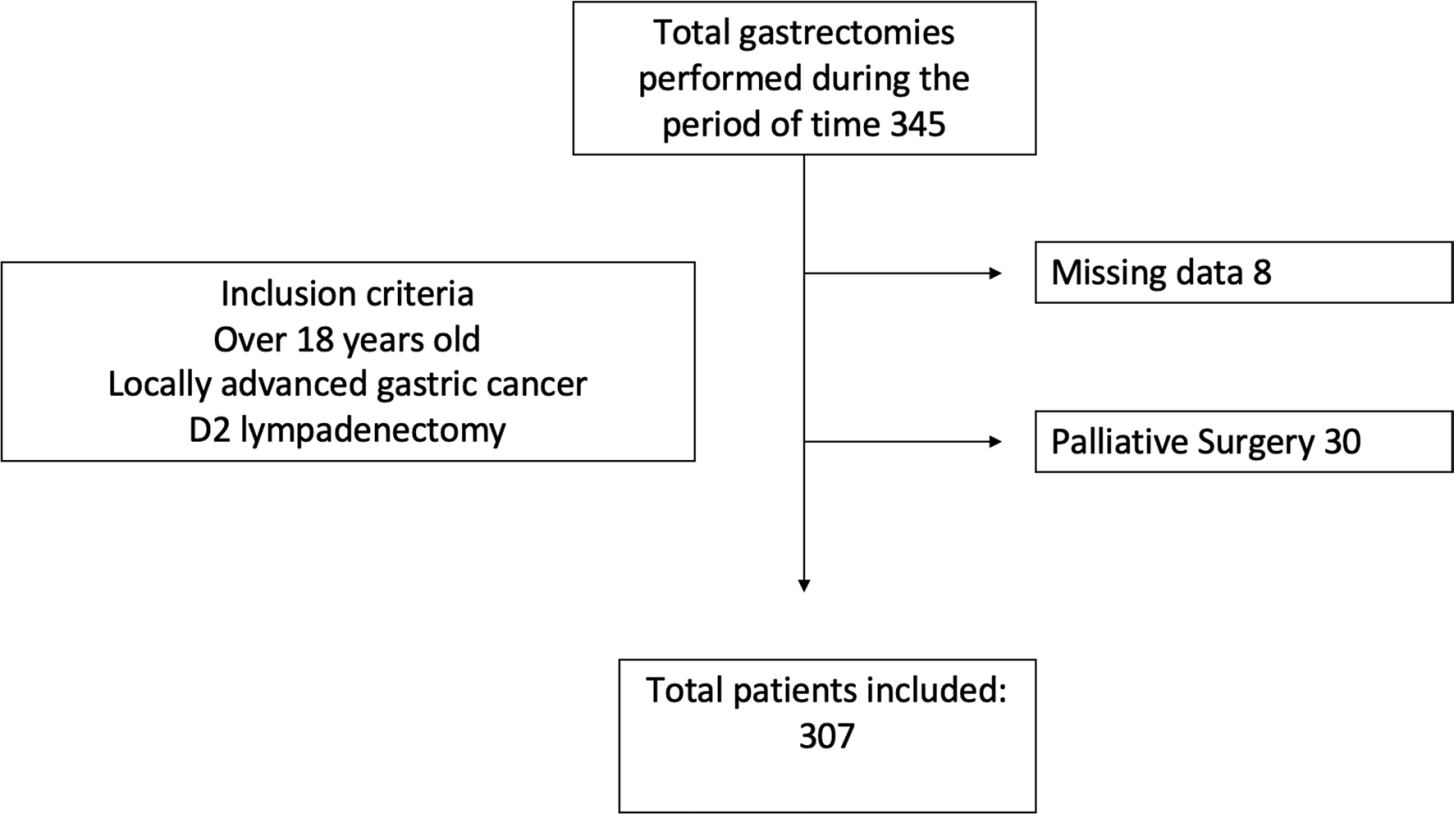
Included patients

All patients with locally advanced gastric cancer in our institution who match inclusion criteria (Fig. 1) were included in our study.

Multidisciplinary teams were requested for all patients with gastric cancer and included nutritional assessment, preoperative boards including radiology, oncology, and nutritional support; when required an intraoperative central catheter was placed for chemotherapy. All patients were diagnosed by esophagogastroduodenoscopy with biopsy confirmation of gastric cancer. To rule out metastases, all patients underwent thoracic and abdominal multidetector contrast computed tomography. All patients underwent staging laparoscopy. Neoadjuvant therapy was considered in all patients. After systemic therapy patients were taken to surgery. All patients included underwent a D2 lymphadenectomy.

Follow up was performed at 15, 30, and 90 days postoperative. Overall survival was estimated according to national databases.

#### Surgical technique

Laparoscopy was assessed by entrance into the abdominal cavity with a 12-mm supraumbilical optical trocar, CO2 pneumoperitoneum, a 12-mm right paramedian trocar, and a 5-mm subxiphoid, left paramedian, and right flank trocar. Complete omentectomy, section of the short vessels with advanced bipolar. Dissection of the right gastroepiploic artery and ligation with polymer clips, dissection of the first portion of the duodenum, identifying gastroduodenal artery, post pyloric duodenal section with 60 mm stapler. a lymphadenectomy of the hepatic artery, hepatoduodenal ligament, splenic artery and splenic hilium was performed. Dissection of the lesser omentum, dissection and ligation of the left gastric artery at the base, en bloc lymphadenectomy of the left gastric artery and the celiac trunk. Dissection of the hiatus and the distal esophagus, section of the esophagus with 60 mm stapler. The esophagoyeyunostomy or gastroyeyunostomy was performed with circular and linear stapler respectively Extraction of surgical piece by enlargement of the umbilical wound with Alexis separator. Thereafter, a section of the jejunum 40 cm from the Treitz ligament with a 60 mm stapler and antecolic loop is raised, opening at the end and entry of a 25 mm circular suture through the incision opening on the right flank. 50 cm distal to the esophagus jejunostomy are measured and laterolateral jejunojejunostomy is performed with 60 mm stapler, closing the defect with seromuscular polydioxanone 3–0. Methylene blue test is always performed. Closure of the hiatus with non-absorbable 2–0 suture, closure of the Petersen defect and intermesenteric with 3–0 polypropylene suture.

## Results

### Patients and operative characteristics

A total of 315 patients underwent gastrectomy for advanced gastric cancer; 8 patients were excluded due to missing data. A total of 307 patients were included in the study. Male patients constituted the majority of the population with 58.63% of the cases (n = 180). The mean age was 60.94 ± 14.51 years old. Not enough information was retrieved to obtain a BMI index. All patients were classified as ASA score 2 and 3. 183 patients received total gastrectomy and 123 subtotal resections. The laparoscopic approach was preferred in 88.93% (n = 273) of the patients; the conversion rate to open surgery was 4.03% due to technical issues. Intraoperative drainage was left in 1 patient. Pre-operative pathological diagnosis was made according to upper endoscopy biopsy. Intestinal adenocarcinoma was the most frequently found in 42.86% (n = 117) of the cases followed by diffuse type in 23.44% (n = 64) of the patients. Signet ring carcinoma type was found in the biopsies of 31.36% (n = 90). Neoadjuvant therapy was administered to 68.40% of the analyzed patients. (n = 210) (Summarized data are displayed in Table 1).

**Table 1:** Patients characteristics and pathological reports

Variable	Value
Male patients % (n)	58.63 (180)
Age mean (SD)	60.94 (14.51)
Total gastrectomy % (n)	59.61 (183)
Laparoscopic	88.58 (163)
Open	11.42 (21)
Subtotal gastrectomy % (n)	40.39 (124)
Laparoscopic	89.43 (109)
Open	10.57 (14)
Conversion rate % (n)	4.03 (11)
Neoadjuvant therapy % (n)	68.40 (210)

Histopathological reports % (n)	
Intestinal Adenocarcinoma	42.86 (117)
Difuse Adenocarcinoma	23.44 (63)
Mixed Adenocarcinoma	5.86 (16)
Neuroendocrine tumors	8.42 (23)
Gist	6.96 (19)
Intraepitelial neoplasia	0.73 (2)
Poor diferenced carcinoma	5.13 (14)
Cistoadenocarcinoma	0.37 (1)
Mucinous	2.93 (8)
Signet ring carcinoma	31.36% (90)

### Postoperative characteristics and outcomes

The overall morbidity rate was 25.91% (n = 71), the most frequent complication was postoperative fistula in 11.72% of the cases (n = 36), and in most of these cases, a leak was evidenced in the esophagojejunostomy (27/36 cases). Other complications analyzed included postoperative bleeding in 2.20% of the patients (n = 6), followed by surgical site infection in 6.23% of the cases (n = 17). Management of postoperative fistula was according to the nature and clinical course of each patient. Initial endoscopic treatment with stent positioning was preferred in 19% (n = 7) of the cases, and reintervention was decided in 27.77% (n = 10) of the patients. Mixed treatment (surgical/endoscopic) was indicated in 41.66% (n = 15) of fistula cases. A complete response (postoperative negative pathology) to the neoadjuvant therapy was observed in 7.49% (n = 23) of the patients. The laparoscopic approach was achieved in 88.58% of total gastrectomies and 89.43% of the subtotal gastrectomies.

A postoperative intensive care unit was required for 24.43% (n = 75) of the cases, with an ICU stay median of 1 day (IQR 1;10). In terms of total in-hospital stay, the median was 5 days (IQR 4;19), and the readmission rate at 30 days of follow-up was 7.81% (n = 24) in most of the cases due to abdominal pain. (Summarized data are displayed in Table 2).

**Table 2:** Postoperative outcomes

Variable	Value
Any complication %(n)	25.91 (71)
Complications	
Fistula	11.72 (36)
Esophago-jejunostomy	27/36
Jejuno-jejunostomy	5/36
Duodenal stump	4/36
Pulmonary embolism	0.37(1)
Enteral perforation	0.73(2)
Surgical site infection	
Superficial	2.20 (6)
Deep	6.23 (17)
Ischemic colitis	0.37(1)
Postoperative ileus	1.10 (3)
Neumonia	2.56 (7)
Splenic ischemia	0.73 (2)
Bleeding	2.20 (6)
Esophago-jejunostomy stenosis	1.47 (4)
Jejuno-jejunostomy stenosis	1.47 (4)
Rates	
Mortality rate	4.03 (11)
Readmission rate	7.81 (24)
Intensive care unit requirement	24.43 (75)
ICU Stay median (IQR)	1 (1;10)
In-hospital length stay	5 (4;19)

Hospital stay was analyzed between surgical approaches. Patients who underwent laparoscopic resection have a lesser mean hospitalization length than the open group. (8.5 vs 13.7 days) with statistically significant value. (p = 0.01). The complication and mortality rates were higher in the laparoscopic group; however, this could be explained by the small sample size of the open approach.

### Oncologic characteristics and survival analysis

Negative margins (R0 resection) were obtained in 93.48% (n = 287) of the patients. The most frequent border compromise was esophageal in 2.93% of patients. Total lymph node retrieval was evaluated, with a median of 20 nodes (IQR 12;37); The median compromised lymph nodes were 4 positive nodes (IQR 0;16). A comparison between lymph node status between open and laparoscopic approaches was performed using the Wilcoxon test. Results demonstrated that the laparoscopic group's lymph node retrieval after surgery was even higher with a statistically significant value (Median 21 vs 12; z = − 2.19, p = 0.02) (see Table 3). There were no statistical differences in the compromised lymph nodes between the groups (Median 4 vs Median 5, z = 0.85, p = 0.32). The 1-year, 3-year and 5-year overall survival rate was 84.04% (n = 258), 66.99% (n = 205), 65.47% (n = 201) respectively. The median overall survival time was 35.4 months (9;124 months). In total survival analysis (5 years follow-up), a comparison between the laparoscopic vs open approach was made at 25% survival time, the mean population overall survival time was 22.83, and in terms of each group, the laparoscopic group shows 25.3 Overall survival months, vs 8.7 months in the open group, a comparison between groups are displayed in Table 4.

**Table 3:** TNM Stage information and Lymph node retrieval

Variables	Categories	n	%
Ring shaped cells	Yes	90	31.36
	No	197	68.64
Complete response	Yes	22	7.72
	No	263	92.28
Section margin	Negative	276	93.56
	Positive esophagus	7	2.37
	Positive gastric	9	3.05
	Positive duodenum	3	1.02
Number of nodes (Media-SD)	23,7	12,75	
Positive nodes (Media-SD)	7,73	7,75	
Staging classification	No data	6	2.27
	IA	38	14.39
	IB	27	10.23
	IIA	53	20.08
	IIB	39	14.77
	IIIA	31	11.74
	IIIB	42	15.91
	IIIC	18	6.82
	IVU	10	3.79

**Table 4:** Comparison between approach

	Open	Laparoscopic
Gender % (n)		
Male	70.58 (24)	57.14 (156)
Female	28.42 (10)	42.85(117)
Age mean (SD)	62.08 (12.44)	60.80 (14.76)
ICU requirement % (n)	44.11(15)	21.97(60)
ICU Stay mean (SD)	1.5 (1.8)	0.7 (1.2)
In hospital stay mean (SD)	8.5 (10.60)	13.73 (15.79)
Morbidity % (n)	2.94 (1)	25.64 (70)
Postoperative fistula	0 (0)	10.62(29)
Mortality	0 (0)	4.02 (11)
Readmision rate % (n)	2.94 (1)	7.69 (21)
R0 resection % (n)	88.2 (30)	93.77 (256)
Total lymph node resection mean (SD)	17.32 (3.51)	21.08 (14.22)
Positive lymph node resected median (SD)	3.29 (5.28)	3.21 (6.19)
Follow up—Months—Median (SD)	36 (12.51)	45 (10.21)
Overall survival 1 year	64.70 (22)	86.08 (235)
Overall survival 3 year	50 (17)	68.86 (188)
Overall survival 5 year	47.05 (16)	67.03 (183)

**Fig. 2:**
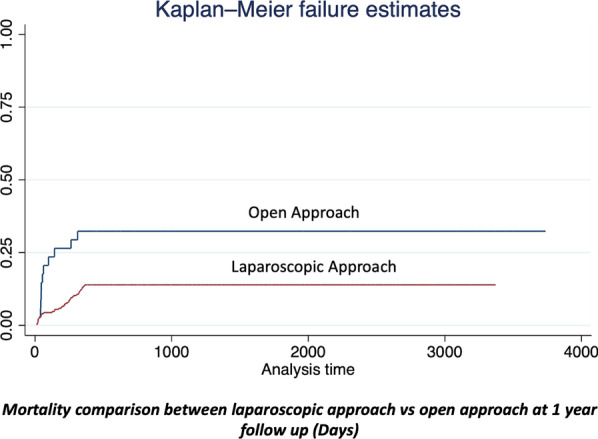
Mortality comparison between laparoscopic approach vs open approach at 1 year follow up (days)

**Fig. 3:**
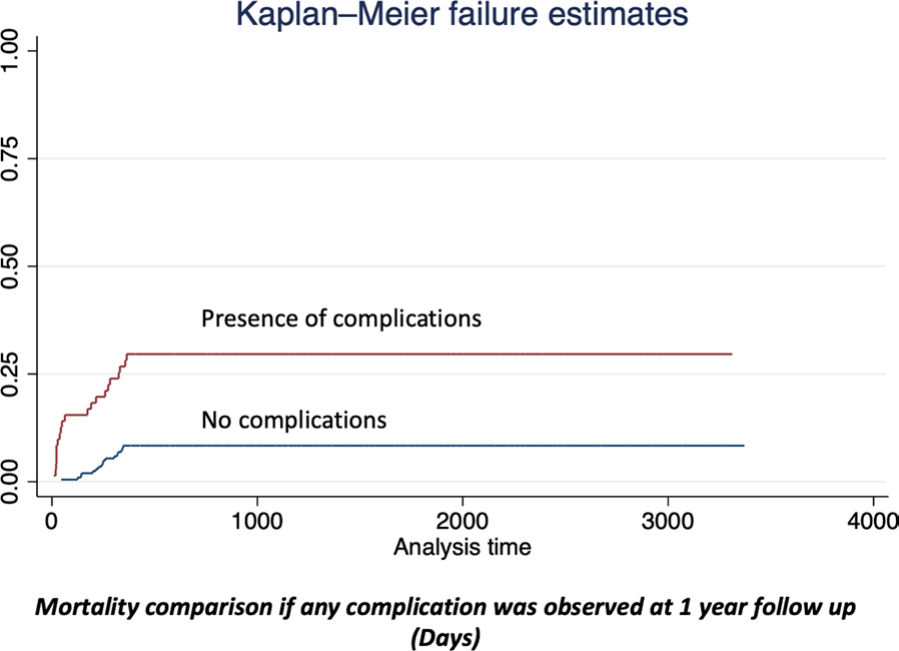
Mortality comparison if any complication was observed at 1 year follow up (days)

In the initial analysis, type of approach, ICU requirement, complication rate, harvested lymph nodes, and readmission were statistically related to 1-year overall survival. In a secondary analysis following a Cox-proportional regression model, the requirement of ICU, presence of any complication, harvested lymph nodes, and readmission rate shows a statistical relationship (see Table 5). Type of approach, total lymph node retrieval, and margins failed to reach statistical value (Figs. 2, 3).

**Table 5:** 1-year overall survival statistical analysis

1 year overall survival	Approach	ICU Requirement	Complication rate	Lymph node retrieval	Harvested lymph nodes	Readmission rate	Resection margins	Signet ring	Neoadjuvance
Log Rank p value (chi2)	0.002 (9.31)	0.000 (43.83)	0.000 (22.34)	0.931 (38.66)	0.000 (69.02)	0.001 (20.37)	0.902 (1.05)	0.241 (1.37)	0.837 (0.04)
Cox proportinal p value (HR)	1.00 (7.5)	0.000 (0.24)	0.000 (2.5)	–	0.009 (1.05)	0.000 (1.5)	–	–	–

**Fig. 4:**
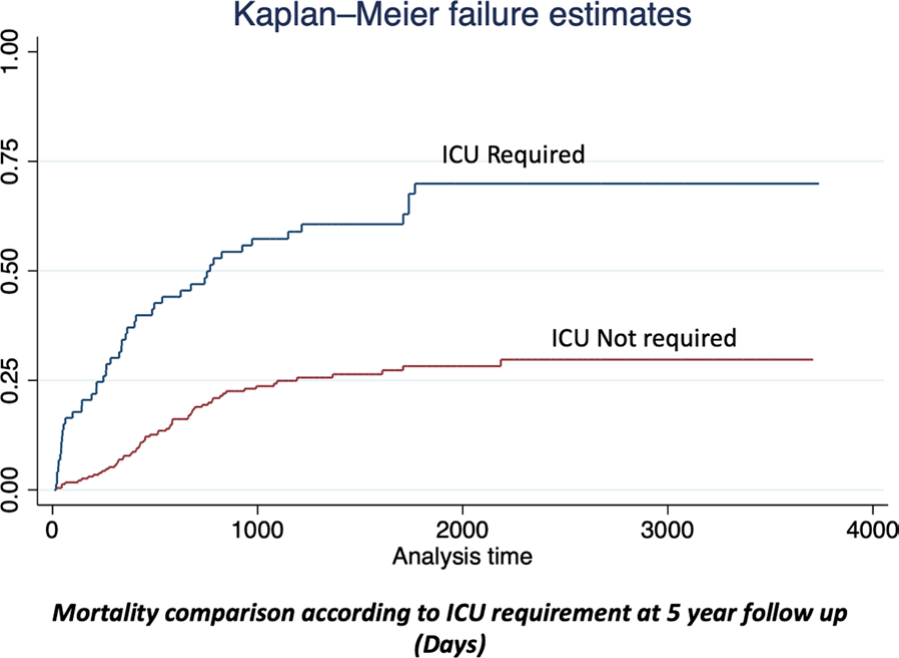
Mortality comparison according to ICU requirement at 5 year follow up (days)

For the 5-year overall survival, the initial log-rank test shows a relationship between the type of approach, ICU requirement, presence of any complication, readmission rates, resection margins, harvested lymph nodes, and total lymph node retrieval and survival after 5 years of follow-up (see Table 6). In the Cox-proportional regression model, only readmission rate, and ICU requirement shows a statistical relationship (Fig. 4).

**Table 6:** 5 Year overall survival statistical analysis

5 year overall survival	Approach	ICU Requirement	Complication rate	Lymph node retrieval	Harvested lymph nodes	Readmission rate	Resection margins	Signet ring	Neoadjuvance
Log Rank p value (chi2)	0.09 (2.80)	0.000 (45.12)	0.012 (6.43)	0.16 (62.75)	0.000 (106.75)	0.001 (21.69)	0.007 (13.96)	0.341 (0.89)	0.471 (0.50)
Cox proportional p value (HR)	1.00 (1.3)	0.000 (0.27)	0.702 (1.09)	0.312 (1.00)	0.001 (1.04)	0.000 (1.49)	0.352 (1.1)	–	–

## Discussion

Notwithstanding the recent and continuous advances in systemic therapy in the treatment of gastric cancer, radical complete resection with a successful and broad lymphadenectomy is a cornerstone for potentially performing a curative treatment [14]. According to international consensus such as ESMO [15] and Japanese guidelines [16], a D2 lymphadenectomy (including: perigastric nodes, named as station from 1 to 6 by the Japanese classification, left gastric: 7th station, common hepatic: 8th station, splenic: 11th station, and coeliac axis arteries: 12th station, with a minimum node dissection of 16 nodes) plus complete resection (R0) are independent prognostic factors for survival outcomes [13, 15, 17].

Diagnostic laparoscopy is recognized as the first step in laparoscopic gastrectomy and it is recommended to use DL in all advanced cases. The agreement of DL with the final stage is high for T stage and M stage (95–98% and 80–100% respectively). Moreover, DL avoids unnecessary laparotomies in approximately 8.5 to 43.8% of the cases [18, 19]. However, in our opinion, this procedure must be used according to individual assessment and not as a routine strategy in all GC patients. Some previous reports based on cost effectiveness recommend to use DL only in cases where the procedure yield is high and some high risk factors are present like (T3, T4 disease, signet ring histology, poor differentiation and lymphadenopathy [20].

Laparoscopic gastrectomy is currently recommended for early gastric cancer treatment by the Japanese gastric cancer association and other clinical guidelines [15, 16]. However, the safety of this approach for advanced gastric cancer (AGC) is controversial. The technical difficulty of minimally invasive surgery has been described previously [14, 21–23]. Deng et al. [13] in a meta-analysis showed that the laparoscopic approach retrieves fewer harvested lymph nodes compared with the open approach (Mean comparison 2.77 vs 4.38 respectively p = 0.0007). In our study, we obtained a median of 20 nodes resected (IQR 12;37) and a difference between the total lymph node retrieval (Median 21 vs Median 12 z = − 2.19, p = 0.02) with statistically significant values between approaches in favor of the laparoscopic approach, according to the international union for cancer control, the examination of 15 nodes is beneficial in gastric cancer [21]. Nevertheless, in terms of compromised lymph nodes, there are no differences between the laparoscopic versus open approach (Median 4 vs Median 5, z = 0.85, p = 0.32).

Since the first use of laparoscopy for AGC by Goh et al. [24], several prospective, multicenter and randomized studies have proved the efficacy and safety of this approach. Most of the studies have been performed in Japan, Korea, and China. However, the application of ACG is quite variable in different countries and regions of the world [11, 15]. In Latin America, information regarding laparoscopic approaches or survival outcomes for AGC is limited [4, 11, 15]. The main reason for the lack of studies is that the technique of laparoscopic gastrectomy remains not standardized. In our institution, given the creation of the minimally invasive group in 2012, the ACG are treated with perioperative systemic therapy and laparoscopic gastrectomy whenever possible. Open gastrectomy was reserved for technically difficult or very large tumors. The surgical technique is standardized among the surgeons of the minimally invasive group.

A meta-analysis published in 2021 compared 6976 LG patients with 7713 in the OG group, regardless of the characteristics of the studies collected (RCT or cohort study), they found a higher frequency of hospital stay and overall/serious complications in the LG than the OG [11]. This data is contrary to our results in which the laparoscopic approach showed a mean difference in hospital length of stay of at least 4 days between groups in favor of LG with a statistically significant value (8.5 vs 13.7 days (p = 0.01). Deng et al. [13] also described a lesser time of in-hospital stay for patients who underwent laparoscopic gastrectomy with a statistically significant value (z =− 1.0 vs z = − 1.83 p 0.02), this data was associated with a less morbidity rate for LG (OR 0.26 CI 95% 0.13–0.54).

Another relevant point to mention is the relation between perioperative systemic therapy and LG. In current guidelines, perioperative systemic therapy is mandatory for AGC. Some previous reports found an increased rate of complications or conversion rates of patients treated with perioperative systemic therapy. In the EORTC trial 40954, the authors described a postoperative complication rate that was higher in the neoadjuvant group than the up front surgery group (27.1% vs. 16.2%; p = 0.09) [25]. A more recent propensity score analysis of a multicenter research on 97 LAGC patients also supports the increased conversion rate of patients previously taken to neoadjuvant therapy and the higher morbidity associated with this scheme. They emphasized the fact that the significance of this difference was only with patients over 60 years old [26]. All of our patients received perioperative systemic therapy so this comparison was not possible to be answered in this research.

Long-term survival analysis was performed in our study; we evidenced a 1-year, 3-year, and 5-year overall survival (OS) rate of 84.04% (n = 258), 66.99% (n = 205), 65.47% (n = 201) respectively. The median overall survival time was 35.4 months (9;124 months). This survival benefit was also described by a retrospective study performed in Italy in 91 patients with a propensity score matching analysis, where the 5-year overall and disease-free survival were higher for patients treated by laparoscopy, but this advantage was significant just in N0 and stages IB and II patients [27–29]. Furthermore, a multicenter randomized clinical (The LOGICA) trial found oncological efficacy similar between laparoscopic and open gastrectomy [7]. This benefit was also identified in the United States, in 2018, Hendricksen et al. in a retrospective propensity score study collected data from 17,449 patients who underwent gastrectomy. The 5 year overall survival benefit of minimally invasive surgery (including laparoscopy and robotic) was superior to open surgery. (51.9% versus 47.7% (P < 0.0001)) [30].

The influence of major postoperative complications and long-term outcomes has been previously described. Li et al. [31] found significant differences in overall survival rates at 5 years of follow-up in patients who present major postoperative complications (46.3% vs. 65.9%, *P* = 0.042) with statistical significance. In the same order, our results demonstrate a statistical relationship between the presence of any complication after surgery and a decrease in the 1-year overall survival; and also, that patients who require ICU stay after surgery have a decreased 5-year overall survival rate with statistical significance, increasing the evidence about the impact of postoperative morbidity after gastrectomy in long-term oncologic outcomes.

Among the limitations of our study includes the retrospective nature, the limited sample size in the open approach leading to imbalances between groups, and the lack of data regarding disease-free survival rate. However, our study increases the evidence in favor of the laparoscopic approach for the treatment of gastric cancer in the Latin-American population and reinforces the importance of the positive outcomes of a high-volume center for the treatment of GC patients.

## Conclusion

According to our data, a laparoscopic approach is a feasible and safe approach with acceptable morbidity and mortality rates for treating gastric cancer. D2 Lymphadenectomy could be performed successfully in a laparoscopic approach in a high-volume center and a properly standardized technique. Major postoperative morbidity with intensive care unit requirement seems to influence overall survival rates. Further prospective studies are needed to confirm our results.

## Data Availability

The datasets used and/or analyzed during the current study are available from the corresponding author upon reasonable request.

## Abbreviations

AGC: Advanced gastric cancer
ICU: Intensive care unit
GC: Gastric cancer
OG: Open radical gastrectomy
LG: Laparoscopic gastrectomy
ICMJE: International Committee of Medical Journal Editors
IQRs: Interquartile ranges
